# Hepatitis B Virus X Protein Driven Alpha Fetoprotein Expression to Promote Malignant Behaviors of Normal Liver Cells and Hepatoma Cells: Erratum

**DOI:** 10.7150/jca.72443

**Published:** 2022-03-02

**Authors:** Mingyue Zhu, Yan Lu, Wei Li, Junli Guo, Xu Dong, Bo Lin, Yi Chen, Xieju Xie, Mengsen Li

**Affiliations:** 1 Hainan Provincial Key Laboratory of Carcinogenesis and Intervention, Hainan Medical College, Haikou 571199, Hainan Province, PR. China.; 2 Key Laboratory of Molecular Biology, Hainan Medical College, Haikou 571199, PR. China.; 3 Department of Pathophysiology, Hainan Medical College, Haikou 571199, Hainan Province, PR. China.; 4 Institution of Tumor, Hainan Medical College, Haikou 570102, Hainan Province, PR. China.

In our paper^1^, the immunohistochemistry assay was applied to detect the expression of CXCR4 in lymph nodes metastasis HCC patients(HBV+)(Figure 1A) was used wrong, so we replace the correct picture in Figure 1A. We are deeply sorry and sincerely apologize for the error and for any inconvenience that may cause to the readers and the editors of this journal. Figure 1A was corrected as follows.

## Figures and Tables

**Figure 1:**
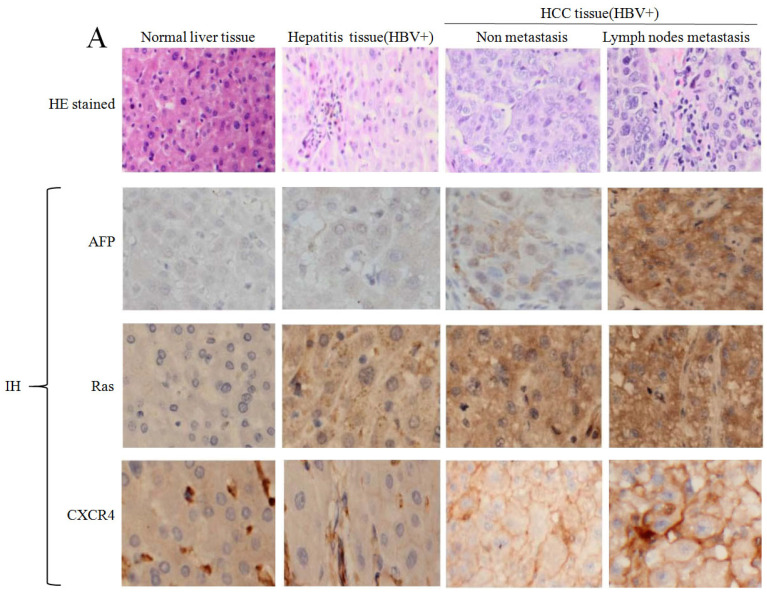
Correlation of HBV with expression of AFP, Ras and CXCR4 in HCC tissues. Clinical liver tissues sample were collected after surgical hepatectomy. A, Expression of AFP, Ras and CXCR4 in the tissues were detected by immunohistochemistry assay. B, Concentration of serum AFP was detected by ELISA. 1, Normal; 2, Hepatitis patients(HBV+); 3, Non metastasis HCC patients(HBV+); 4, Lymph nodes metastasis HCC patients(HBV+). C, Expression of AFP, Ras and CXCR4 in the tissues were detected by Western blotting, right column images represented the proteins densitometry value ratio compared with internal control β-actin. 1, Normal liver tissues; 2, Hepatitis tissues(HBV+); 3, Non metastasis HCC tissues(HBV+); 4, Lymph nodes metastasis HCC tissues(HBV+). The images representation of at last three reduplicate experiments. HE stained: Haematoxylin and eosin stained; IH: Immunohistochmeistry stained.
